# Akt Phosphorylates Both Tsc1 and Tsc2 in Drosophila, but Neither Phosphorylation Is Required for Normal Animal Growth

**DOI:** 10.1371/journal.pone.0006305

**Published:** 2009-07-17

**Authors:** Sibylle Schleich, Aurelio A. Teleman

**Affiliations:** German Cancer Research Center (DKFZ), Heidelberg, Germany; University of Texas MD Anderson Cancer Center, United States of America

## Abstract

Akt, an essential component of the insulin pathway, is a potent inducer of tissue growth. One of Akt's phosphorylation targets is Tsc2, an inhibitor of the anabolic kinase TOR. This could account for part of Akt's growth promoting activity. Although phosphorylation of Tsc2 by Akt does occur in vivo, and under certain circumstances can lead to reduced Tsc2 activity, the functional significance of this event is unclear since flies lacking Akt phosphorylation sites on Tsc2 are viable and normal in size and growth rate. Since Drosophila Tsc1, the obligate partner of Tsc2, has an Akt phosphorylation motif that is not conserved in mammals, we investigate here whether Akt redundantly phosphorylates the Tsc complex on Tsc1 and Tsc2. We provide evidence that Akt phosphorylates Tsc1 at Ser533. We show that flies lacking Akt phosphorylation sites on Tsc1 alone, or on both Tsc1 and Tsc2 concurrently, are viable and normal in size. This shows that phosphorylation of the Tsc1/2 complex by Akt is not required for Akt to activate TORC1 and to promote tissue growth in Drosophila.

## Introduction

The protein complex consisting of Tsc1 (also known as hamartin) and Tsc2 (also known as tuberin) has emerged in the past decade as an important regulator of the potent anabolic kinase TOR complex 1 (TORC1) (for review see [1]). The Tsc1/2 complex appears to sense a large number of inputs such as the presence of growth factors, cytokines, energy stress and hypoxia, and integrates this information to regulate the activity of TORC1 via the GTPase Rheb [1]. TORC1 in turn regulates cellular translation rates to affect both cell growth (and consequently organismal size) and metabolism [2]–[4]. This ‘signaling cassette’ is highly conserved in evolution, and many of the discoveries piecing together the molecular connections between components of this cassette were concurrently performed in multiple model systems such as Drosophila and mice, leading to equivalent results.

One function of the Tsc1/2 complex appears to be to mediate the activation of TORC1 in response to Akt. The current model proposes that in response to insulin/IGF signaling, PI3K and subsequently Akt become activated. Upon activation, Akt phosphorylates Tsc2 on numerous sites. This inactivates the Tsc1/Tsc2 complex, relieving the suppression of TORC1 by Tsc1/2, leading to TORC1 activation and cell growth. This would provide a molecular link by which insulin-mediated activation of Akt leads to TORC1 activation, and hence tissue growth. However, the in vivo relevance of this function for Tsc1/2 is unclear due to discordant findings in the literature. This model is supported by a large body of evidence. In both mammalian systems and in flies, Tsc2 is indeed phosphorylated by Akt in vivo and in vitro [5]–[7]. The model predicts that alanine-substitution mutants of Tsc2 lacking the Akt phosphorylation sites should be insensitive to Akt activity. Indeed, overexpression of such mutants leads to a more powerful suppression of TORC1 activity compared to overexpression of wildtype Tsc2 [5]–[8], and this overexpression is able to dominantly block Akt-mediated activation of TORC1 [5]–[8]. This is the case in mammalian cell culture, Drosophila cell culture as well as in Drosophila tissues, and indicates that at least when Tsc2 is overexpressed, the ability of Akt to phosphorylate it is functionally relevant. The most rigorous test, however, to check whether the phosphorylation of Tsc2 by Akt is functionally important for an animal is to generate mutant animals in which endogenous Tsc2 is replaced by a non-phosphorylatable alanine-substitution mutant. This experiment, asking what happens when Tsc2 cannot be phosphorylated by Akt in vivo, was performed by Dong and Pan in 2004 [9]. They generated flies in which they mutated the endogenous Tsc2 gene and simultaneously expressed either a wildtype Tsc2 or a mutant Tsc2 in which all four Akt phosphorylation sites were mutated to alanine or to a phosphomimetic residue. Surprisingly, although Tsc2 null flies, like mice, die early in development, flies containing either alanine-substitution or phosphomimicking mutants of Tsc2 were viable, fertile, normally patterned and normal in size and growth rate [9]. This suggests that at least in Drosophila, although Akt can and does phosphorylate Tsc2 on multiple sites, this phosphorylation is functionally not very important.

An open question is how to interpret this result and to reconcile it with the remaining body of evidence mentioned above. Is phosphorylation of Tsc2 by Akt important for Akt to drive tissue growth in vivo or not? One option is that the result by Dong and Pan reflects something specific to Drosophila. Indeed, as was noted previously [7], Drosophila Tsc1 - the binding partner of Tsc2 - also contains a consensus Akt phosphorylation site (Ser533) which is not conserved in mammals. Both Tsc1 and Tsc2 need to be active to achieve normal activity of the complex, and recently it has been shown that phosphorylation of Tsc1 (e.g. by IKKβ, [10]) can inhibit Tsc1/2 complex activity in cell culture. Thus it is possible that Akt phosphorylates both partners of the Tsc1/2 complex in Drosophila, and that unless phosphorylation of both partners is simultaneously abrogated, Akt will be able to disrupt Tsc1/2 function. This possibility is strengthened by the fact that Ser533 is reported to be phosphorylated in vivo in Drosophila KC167 cells, detected by mass spectroscopy (www.phosphopep.org [11]).

In this study, we examine whether Tsc1 is phosphorylated by Akt in Drosophila, and the physiological consequences of this phosphorylation. We provide evidence that Akt phosphorylates Tsc1 at Ser533 and that this phosphorylation is induced by insulin signaling. We test genetically the requirement for this phosphorylation by engineering Tsc1 mutants in which the Akt phosphorylation sites are mutated to nonphosphorylatable (Tsc1^S533A^) or phosphomimetic (Tsc1^S533D^) residues, and show that in both cases the flies are rescued to full viability and size. To ask whether the phosphorylation of dTsc1 and dTsc2 by Akt are functionally redundant, we genetically engineer flies in which both Tsc1 and Tsc2 are simultaneously replaced with mutant versions that cannot be phosphorylated by Akt (Tsc1^S533A^, Tsc2^T437A/S924A/T1054A/T1518A^). Surprisingly, these animals are also viable and normal in size and growth rate. This shows that phosphorylation of both Tsc1 and Tsc2 is not required for Akt to drive tissue growth in Drosophila, indicating that other targets of Akt must be responsible for Akt's growth-promoting activity. We do find, nonetheless, that these animals have mild metabolic defects, raising the possibility that the regulation of Tsc1/2 by Akt plays a fine-tuning role in organismal metabolism. This would be similar to what is seen with other components of the pathway, such as Rictor and Melted, which play important yet modulatory functions during animal development [12], [13].

## Results

### Akt phosphorylates dTsc1 on Ser533

**Figure 1:**
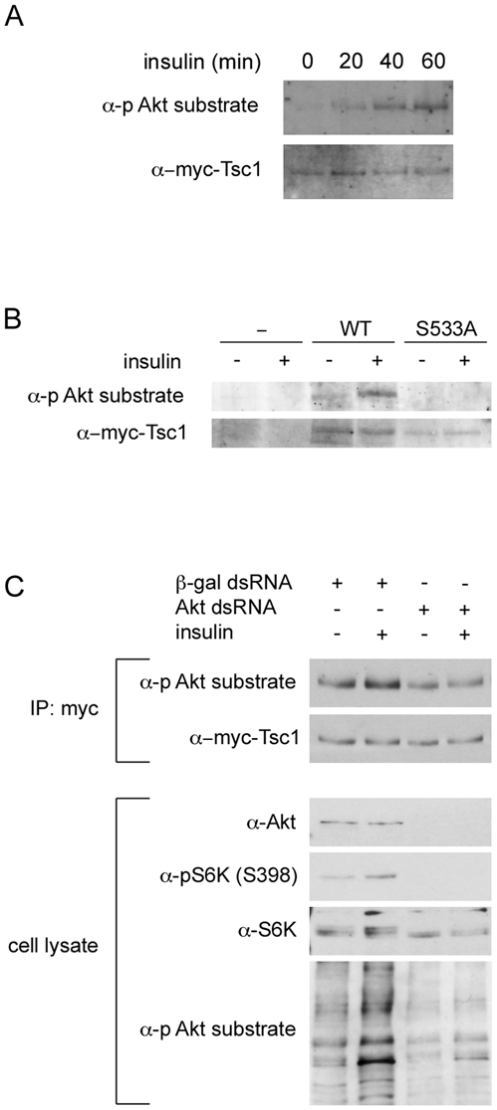
Drosophila Tsc1 is phosphorylated by Akt on Ser533. (A) Phosphorylation of Tsc1 increases with insulin treatment. S2 cells transfected with constructs to express myc-Tsc1 and His-Tsc2 were treated without insulin (0 min) or with insulin (10 µg/mL) for indicated times (20, 40 or 60 min). Cells were then lysed and myc-Tsc1 immunoprecipitated using anti-myc antibody. Immunoprecipitates were probed with anti-myc antibody as a loading control, and anti-Phospho-(Ser/Thr) Akt Substrate antibody to detect phosphorylation of Tsc1. (Ser533 is part of an Akt phosphorylation consensus motif). (B) Tsc1 is phosphorylated on Ser533 in response to insulin treatment. Untransfected S2 cells (-) or S2 cells transfected with constructs to express either myc-Tsc1^WT^ (“WT”) or myc-Tsc1^S533A^ (“S533A”) together with His-Tsc2 were treated with or without insulin (10 µg/mL) for 1 hour prior to lysis and immunoprecipitation with anti-myc antibody. Immunoprecipitates were probed with anti-myc antibody as a loading control, and anti-Phospho-(Ser/Thr) Akt Substrate antibody to detect phosphorylation of Tsc1. (C) Knockdown of Akt abrogates the increase in phosphorylation of Tsc1 on Ser533 induced by insulin treatment. S2 cells transfected with expression constructs for myc-Tsc1^WT^ and His-Tsc2 were treated with control dsRNA or Akt dsRNA for 4 days prior to insulin treatment (10 µg/mL for 1 hour), lysis and anti-myc immunoprecipitation. Immunoprecipitates were probed with anti-myc as a control and anti Phospho-(Ser/Thr) Akt Substrate antibody to detect phosphorylation of Tsc1 Ser533. Despite efficient knockdown of Akt (seen by lack of Akt protein and S6K phosphorylation in lanes 3 and 4), anti Phospho-(Ser/Thr) Akt Substrate antibody displays background binding in total cell lysates, as previously reported also by others.

As was previously noted [7], Drosophila Tsc1 contains a perfect Akt phosphorylation consensus (R-x-R-x-x-S/T) at Ser533 – RNRMAS – which is not conserved in mammalian Tsc1 proteins. This raises the possibility that Akt phosphorylates dTsc1 in addition to dTsc2 in Drosophila. Furthermore, using a proteomic approach in which phosphopeptides were identified from extracts of Drosophila Kc167 cells using mass spectroscopy, Aebersold and colleagues have detected phosphorylation on Ser533 of endogenous Tsc1, indicating that this site is phosphorylated in vivo by an unknown kinase (www.phosphopep.org [11]). Therefore, we decided to test if Akt phosphorylates dTsc1 at Ser533. To detect phosphorylation at this site, we transfected S2 cells with a construct expressing myc-tagged Tsc1. We then immunoprecipitated Tsc1 using the myc tag, and detected phosphorylation using the Phospho-(Ser/Thr) Akt Substrate antibody from Cell Signaling which recognizes (R-x-R-x-x-phosphoS/T) epitopes. This antibody should recognize Ser533 on Tsc1 if it is phosphorylated. When S2 cells were transfected to express wildtype Tsc1, a weak phospho-signal could be detected in the myc-Tsc1 immunoprecipitate (Figure 1A, Lane 1) which increased progressively in strength when the cells were treated with insulin for 20, 40 or 60 minutes prior to lysis (Figure 1A, Lanes 2–4), indicating that this phosphorylation is insulin responsive. We then tested which site on Tsc1 is responsible for this signal. When S2 cells were transfected to express wildtype myc-Tsc1, the signal in the anti-myc immunoprecipitate increased in strength upon insulin treatment, consistent with the previous result (Figure 1B, lanes 3 and 4). This signal was not detectable if cells were not transfected, indicating the phospho-specific antibody is specifically detecting myc-Tsc1 in the myc-IP (Figure 1A, lanes 1 and 2). Furthermore, no phospho-specific signal was detectable if we transfected a myc-tagged Tsc1 in which Ser533 was mutated to alanine, indicating that the phospho-specific antibody is specifically recognizing phosphorylation on Ser533 (Figure 1, lanes 5 and 6). Together, these data suggested that Tsc1 is phosphorylated on Ser533 by a kinase that is activated in response to insulin signaling. Since Ser533 lies within an Akt phosphorylation motif, and since Akt is activated upon insulin stimulation, a likely candidate for this kinase is Akt.

To ask whether the increased phosphorylation of Ser533 on dTsc1 in response to insulin is due to Akt, we tested whether knockdown of Akt was able to blunt this response. We transfected S2 cells with myc-Tsc1(WT), treated the cells with dsRNA against Akt or a control, and then treated the cells with or without insulin before detecting phosphorylation on Tsc1. As shown in Figure 1C, insulin treatment caused an increase in Tsc1 phosphorylation in control cells (lanes 1 and 2), but not in cells treated with Akt dsRNA (lanes 3 and 4). As observed also by others [13], the Phospho-(Ser/Thr) Akt Substrate antibody from Cell Signaling shows a background signal (both top and bottom panel, Fig. 1C). This background signal, most clearly visualized when probing total cell lysates (bottom panel, Figure 1C) is retained when Akt is completely knocked-down, as controlled with anti-Akt antibody and by loss of S6K phosphorylation (Fig. 1C). Since the banding pattern visible when cell lysates are probed with this antibody are similar in the presence and absence of Akt (bottom panel Fig. 1C), this likely reflects residual binding of the antibody to non-phosphorylated R-x-R-x-x-S/T motifs. This has also been reported by others (Figure 1G in [13]). Despite the background signal, the fact that the phospho-signal on Tsc1 no longer increases upon insulin treatment when Akt is removed, indicates Akt is responsible for the insulin-induced phosphorylation of dTsc1 at Ser533.

### dTsc1 phosphorylation by Akt is dispensable in vivo in Drosophila

Some groups have reported that binding between dTsc1 and dTsc2 depends on phosphorylation of Tsc2 [7], whereas others have reported that it does not [9]. We also could not detect any changes in dTsc1/dTsc2 binding in the presence or absence of insulin (not shown), so we could not use a binding assay to probe the effect of Tsc1 phosphorylation on Tsc1/2 function. Therefore we decided to move to an in vivo model.

**Figure 2:**
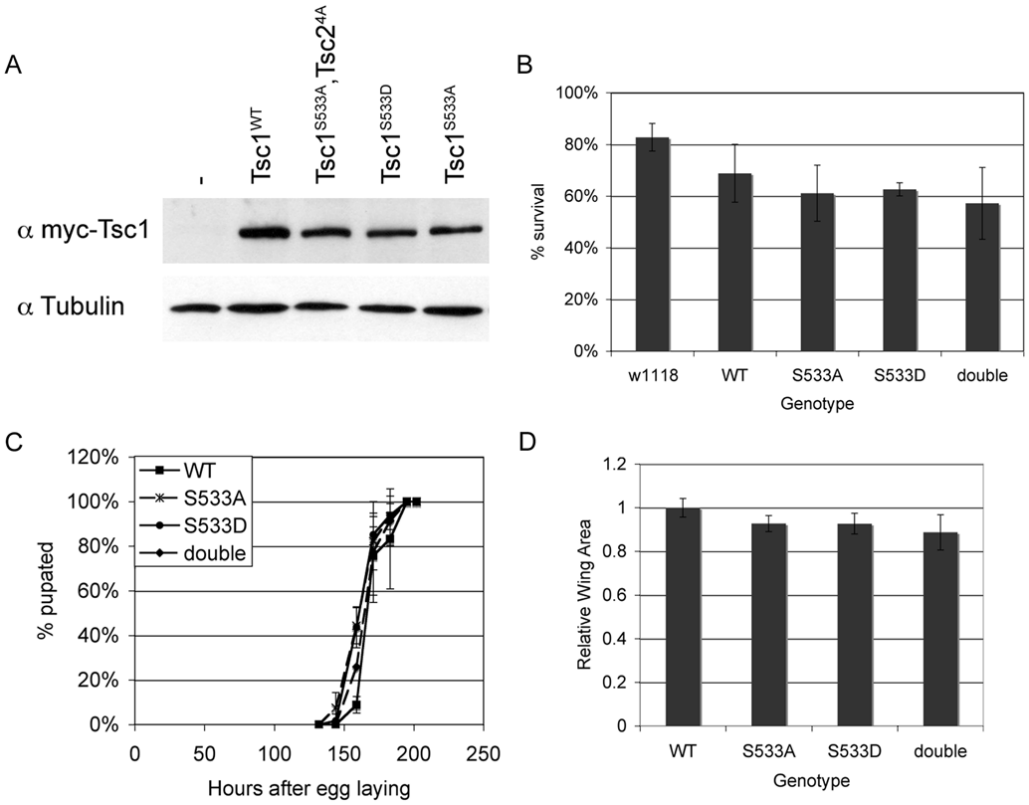
Flies lacking Akt phosphorylation sites on Tsc1 and Tsc2 are viable and normal in size. (A) Expression levels of myc-Tsc1 in fly lines homozygous for the Tsc1^29^ mutation, rescued by ubiquitous expression of Tsc1^WT^, Tsc1^S533A^ or Tsc1^S533D^, or flies homozygous for both the Tsc1^29^ and Tsc2^192^ mutations rescued to viability by ubiquitous expression of both Tsc1^S533A^ and Tsc2^T437A/S924A/T1054A/T1518A^ (“Tsc1^S533A^,Tsc2^4A^”). (B,C,D) Survival rates (B), pupation curves (C) and relative adult wing sizes (D) of animals seeded as L1 larvae under controlled growth conditions for genotypes w^1118^ (“w^1118^”), Tsc1^29^ homozygotes rescued by ubiquitous expression of Tsc1^WT^ (“WT”), Tsc1^S533A^ (“S533A”) or Tsc1^S533D^(“S533D”), or flies homozygous for both the Tsc1^29^ and Tsc2^192^ mutations rescued to viability by ubiquitous expression of both Tsc1^S533A^ and Tsc2^T437A/S924A/T1054A/T1518A^ (“double”).

To test the physiological relevance of this phosphorylation event in vivo, we genetically engineered flies in which endogenous Tsc1 was replaced with various mutant versions. To achieve this, we generated transgenic flies ubiquitously expressing either wildtype Tsc1 (Tsc1^WT^), or Tsc1 variants where Ser533 was mutated to non-phosphorylatable alanine (Tsc1^S533A^) or to a phosphomimicking residue (Tsc1^S533D^). These transgenes were then crossed into a Tsc1^29^ mutant background, in a manner similar to that done by Dong and Pan for Tsc2 [9]. The Tsc1^29^ mutation replaces amino acid 61 with a stop codon, truncating most of the protein, leading to a predicted null [14]. While Tsc1^29^ mutant animals die very early around the embryo-larval transition [14], presence of the Tsc1^WT^ transgene was able to rescue them to adulthood, generating a viable stock with no obvious defects. By picking first instar larvae and seeding them at fixed density on standard flyfood, we found that 83% of control w^1118^ larvae survived to adulthood, and 69% of Tsc1^29^ mutants were rescued to adulthood with the Tsc1^WT^ transgene (Figure 2B, “WT”). We then chose transgenes expressing Tsc1^S533A^ or Tsc1^S533D^ at levels similar to Tsc1^WT^ (Figure 2A, lanes 2, 4 and 5), introduced them into the Tsc1^29^ background, and found that they were also able to rescue the mutant flies as efficiently as the Tsc1^WT^ construct: 61% and 63% of Tsc1^29^ mutants were rescued to adulthood with the Tsc1^S533A^ and Tsc1^S533D^ transgenes respectively (Figure 2B). Furthermore, flies rescued by the wildtype and two mutant constructs showed similar developmental timing, gauged by pupation curves (Figure 2C), and similar final animal size, measured by wing area (Figure 2D). Although wing size was mildly reduced in both Tsc1^S533A^ and Tsc1^S533D^ flies compared to Tsc1^WT^ flies, opposite effects would be expected from the alanine-substitution and phosphomimicking transgenes, making it unclear if this mild reduction is of significance. In sum, the ability of all three transgenes to rescue Tsc1^29^ mutants from early lethality to adulthood suggests that phosphorylation of Tsc1 by Akt is not critical for normal development in Drosophila.

### Flies simultaneously lacking Akt phosphorylation of Tsc1 and Tsc2 are viable but have mild metabolic defects

The Tsc1 and Tsc2 proteins work together as a complex to achieve maximal activity, and recent reports indicate that phosphorylation of either Tsc1 or Tsc2 can lead to regulation of the complex in cell culture [1], [10]. To test whether phosphorylation by Akt of Tsc1 and Tsc2 might be acting redundantly, we generated flies in which both endogenous Tsc1 and Tsc2 were simultaneously replaced with alanine-substitution mutants (tsc1^29^, gig^192^, Tsc1^S533A^, Tsc2^T437A/S924A/T1054A/T1518A^) (gig^192^; Tsc2^T437A/S924A/T1054A/T1518A^ flies kindly provided by D. Pan [9]). To our surprise, these animals were also viable (Figure 2B, “double”), and had similar growth rates and final size compared to animals harboring the Tsc1^WT^ transgene (Figures 2C and 2D, “double”). This is in stark contrast to animals lacking Tsc1, Tsc2, Akt, or Rheb, all of which are lethal early in development [14]–[18]. This indicates that even if the ability of Akt to phosphorylate both partners of the Tsc1/2 complex is abrogated, flies are quite normal in terms of growth, and that in Drosophila the ability of Akt to drive tissue growth does not depend strongly on the Tsc1/2 complex.

**Figure 3:**
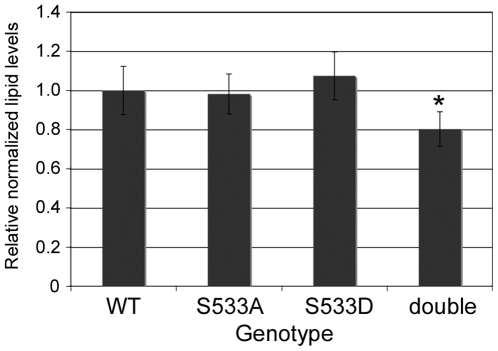
Flies lacking Akt phosphorylation sites on both Tsc1 and Tsc2 are slightly lean. Triglyceride levels normalized to total body protein for Tsc1^29^ homozygotes rescued by ubiquitous expression of Tsc1^WT^ (“WT”), Tsc1^S533A^ (“S533A”) or Tsc1^S533D^(“S533D”), or flies homozygous for both the Tsc1^29^ and Tsc2^192^ mutations rescued to viability by expression of both Tsc1^S533A^ and Tsc2^T437A/S924A/T1054A/T1518A^ (“double”). * indicates statistical significance (ttest = 0.01).

Both insulin signaling and TORC1 are known to regulate animal metabolism in addition to growth. Indeed, flies mutant for a number of components of the pathway, such as rictor or melted, display very mild growth impairments, but have strong metabolic defects [12], [13], [19]. This suggests that animal metabolism is more sensitive to TOR activity than animal growth. Therefore, in order to not overlook more mild defects, we tested whether phosphorylation of the Tsc1/2 complex by Akt might affect organismal metabolism by measuring animal lipid levels. Although animals in which endogenous Tsc1 was replaced with either Tsc1^S533A^ or Tsc1^S533D^ did not reproducibly show alterations in lipid levels (Figure 3), animals in which both Tsc1 and Tsc2 were simultaneously replaced with alanine-substitution mutants were mildly leaner than controls (Figure 3, “double” vs “WT”, ttest = 0.01). This suggests that phosphorylation of the Tsc1/2 complex by Akt might possibly be involved in the more subtle regulation of animal metabolism, as is seen with other modulators of the pathway such as Rictor or Melted [12], [13], [19].

## Discussion

### dAkt phosphorylates dTsc1

We present evidence here that Drosophila Tsc1 is phosphorylated on Ser533 by Akt. Although this serine is conserved in mouse and human Tsc1, the R-x-R-x-x-S motif is not conserved. (In human Tsc1 the respective sequence is 519-THSAAS-524). Since Akt does not absolutely require the full R-x-R-x-x-S motif to recognize its targets [20], we tested whether human Tsc1 is also phosphorylated on Ser524, but could not detect any phosphorylation by mass spectroscopy with immunopurified hTsc1 from HEK293 cells (data not shown). Therefore, we believe this is likely a Drosophila-specific phosphorylation. This phosphorylation site is in very close proximity to Ser487 and Ser511 of human Tsc1, which were recently shown to be phosphorylated by IKKβ [10], leading to regulation of Tsc1/2 function in cell culture. Therefore, it is possible that this clustering of phosphorylation sites in one region of Tsc1 is of functional significance, in particular since it is close to the domain that interacts with Tsc2.

### Is phosphorylation of the Tsc1/2 complex by Akt important?

The finding by Dong and Pan, that flies lacking Akt phosphorylation sites on Tsc2 are viable and normal in size, was surprising [9]. Since we found here that Akt also phosphorylates Tsc1 in Drosophila, this raised the possibility that the phosphorylation of Tsc1 and Tsc2 by Akt are functionally redundant, and that a phenotype is only revealed when both are abrogated. However, to our surprise, we found that flies simultaneously lacking Akt phosphorylation sites on both Tsc1 and Tsc2 are also viable and almost normal in size, reinforcing the conclusion that the connection from Akt to TOR via the Tsc1/2 complex is not critical for normal size and growth. Since Akt strongly activates TORC1 activity and induces tissue growth, this suggests other targets of Akt must be responsible for these effects. Recently, PRAS40 has also been suggested to link Akt to TOR: some groups have reported that Akt can phosphorylate PRAS40, thereby relieving the inhibition of TOR by PRAS40 [21], [22]. Although other groups have reported conflicting data, or alternate interpretations of this data [23]–[25], it is possible that Akt activates TOR via both Tsc1/2 and PRAS40 in a redundant manner, or that other unknown links between Akt and TOR exist. This redundancy would generate a more ‘robust’ system in which TORC1 activity is held in check by two independent pathways, both of which are downstream of Akt. Furthermore, a number of inputs regulate activity of the Tsc1/2 complex, phosphorylation by Akt being only one of them.

One interpretation of our data is that abrogation of the ability of Akt to phosphorylate the Tsc1/2 complex has no functional consequences whatsoever for the animal. Since we find this hard to believe, we tested whether there might be more mild defects in the mutant flies. TOR regulates both tissue growth and organismal metabolism. Some mutations in the fly with mild effects on TOR activity cause small or negligible alterations in animal size, but significant alterations in metabolic parameters such as total body lipid levels [12], [13]. This suggests that metabolic regulation is more sensitive to TOR activity than animal size. Therefore, we tested whether flies simultaneously lacking Akt phosphorylation sites on Tsc1 and Tsc2 are metabolically normal. Indeed, we found that these flies have a mild reduction in body lipid levels. Therefore it is possible that the link between Akt and TOR via the Tsc1/2 complex is more important for fine-tuning animal metabolism than for controlling animal growth.

## Materials and Methods

### Molecular Biology & Fly stocks

Vectors for expressing myc-tagged dTsc1 and His/V5-tagged Tsc2 under control of the actin promoter were a kind gift from Duojia Pan [14]. Point mutations in TSC1 at serine 533 were introduced by PCR to obtain an alanine mutant (oligo GAACCATTTCCACTGTAggcTGCCATACGATTGCG), or an aspartic acid mutant (oligo GAACCATTTCCACTGTAgtcTGCCATACGATTGCG) Final constructs were resequenced to confirm presence of the mutations. To generate transgenic flies, Tsc1-WT, Tsc1-S533A and Tsc1-S533D were subcloned into a pCasper4-based vector containing a tubulin promotor and an SV40 polyA. Flies containing the gigas^192^ mutation, expressing either wildtype Tsc2 or Tsc2^T437A/S924A/T1054A/T1518A^ were a kind gift from Duojia Pan [9].

### Cell culture, Immunoprecipitations and Antibodies

Transfections of Tsc1 and Tsc2 constructs were carried out in S2 cells grown in SFM medium using Cellfectin Reagent (Invitrogen). Twenty hours after transfection, cells were treated with or without bovine insulin for one hour (10 µg/mL, Sigma), then lysed in lysis buffer (50 mM Tris-pH 7.5, 150 mM NaCl_2_, 1% Triton X-100), containing protease and phosphatase inhibitors (Roche). Immunoprecipitations were performed using rabbit anti myc antibody from Cell Signaling (#71D10), and Protein-A agarose beads (Roche). dsRNA targeting both AKT isoforms was generated using oligos containing T7 promoter sequences fused to GAGATTGTGTGTGTTTCGT or GTTCCGGAATCGTGTGTA. dsRNA was added to the medium at 12 µg/mL for 4 days.

Antibodies: anti p-Thr398 dS6k (Cell Signaling, #9209), anti-AKT (Cell Signaling #9272), anti myc (Dianova MA1-980), anti-tubulin (DS Hybridoma Bank AA4.3-s), anti dS6K (kind gift from Mary Stewart).

### Fat measurement

Age and nutrient controlled flies were collected three or five days after hatching and subjected to fat measurement as previously described [19]. Samples of at least five flies were homogenized in ice-cold homogenization buffer (0.05% Tween 20 in H2O). A sample was kept for Bradford protein measurement and assayed immediately. The remaining homogenate was heat-inactivated at 70°C for 5 min. After addition of lipoprotein lipase (Sigma 62333, final concentration 0.25 µg/µl, 37 degrees, overnight) glycerol from the reaction was quantified using Free Glycerol Reagent (Sigma F6428). Experiments were done in quintuplicate.
